# *Aedes aegypti* abundance in urban neighborhoods of Maricopa County, Arizona, is linked to increasing socioeconomic status and tree cover

**DOI:** 10.1186/s13071-023-05966-z

**Published:** 2023-10-08

**Authors:** Jenna E. Coalson, Danielle M. Richard, Mary H. Hayden, John Townsend, Dan Damian, Kirk Smith, Andrew Monaghan, Kacey C. Ernst

**Affiliations:** 1 Mel and Enid Zuckerman College of Public Health, University of Arizona, Tucson, AZ USA; 2 Lyda Hill Institute for Human Resilience, University of Colorado, Colorado Springs, CO USA; 3 Maricopa County, Environmental Services Department, Vector Control Division, Phoenix, AZ USA; 4 University of Colorado, Boulder, CO USA

**Keywords:** *Aedes aegypti*, Desert, Microclimate, Land cover, Coupled human-natural systems

## Abstract

**Background:**

Understanding coupled human-environment factors which promote *Aedes aegypti* abundance is critical to preventing the spread of Zika, chikungunya, yellow fever and dengue viruses. High temperatures and aridity theoretically make arid lands inhospitable for *Ae. aegypti* mosquitoes, yet their populations are well established in many desert cities.

**Methods:**

We investigated associations between socioeconomic and built environment factors and *Ae. aegypti* abundance in Maricopa County, Arizona, home to Phoenix metropolitan area. Maricopa County Environmental Services conducts weekly mosquito surveillance with CO_2_-baited Encephalitis Vector Survey or BG-Sentinel traps at > 850 locations throughout the county. Counts of adult female *Ae. aegypti* from 2014 to 2017 were joined with US Census data, precipitation and temperature data, and 2015 land cover from high-resolution (1 m) aerial images from the National Agricultural Imagery Program.

**Results:**

From 139,729 trap-nights, 107,116 *Ae. aegypti* females were captured. Counts were significantly positively associated with higher socioeconomic status. This association was partially explained by higher densities of non-native landscaping in wealthier neighborhoods; a 1% increase in the density of tree cover around the trap was associated with a ~ 7% higher count of *Ae. aegypti* (95% CI: 6–9%).

**Conclusions:**

Many models predict that climate change will drive aridification in some heavily populated regions, including those where *Ae. aegypti* are widespread. City climate change adaptation plans often include green spaces and vegetation cover to increase resilience to extreme heat, but these may unintentionally create hospitable microclimates for *Ae. aegypti*. This possible outcome should be addressed to reduce the potential for outbreaks of *Aedes*-borne diseases in desert cities.

**Graphical Abstract:**

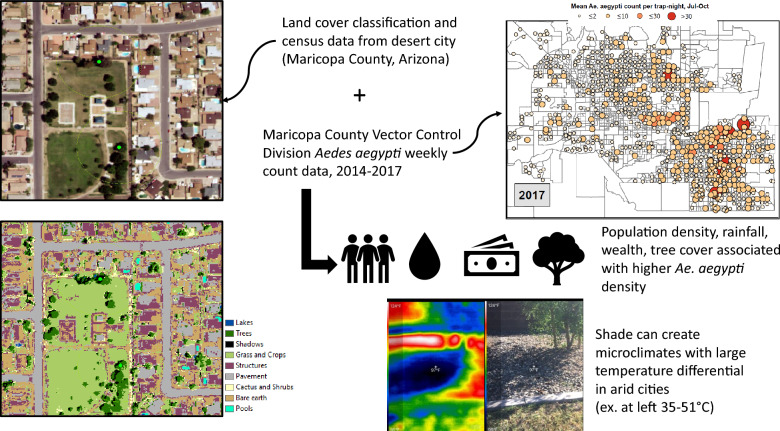

**Supplementary Information:**

The online version contains supplementary material available at 10.1186/s13071-023-05966-z.

## Background

*Aedes aegypti* mosquitoes are key vectors for dengue, chikungunya, Zika, and yellow fever viruses. These mosquitoes thrive in urban environments. They are highly anthropophilic and exploit small breeding sites near human habitation. They are also highly adaptable, developing the ability to exploit previously uninhabited niches, such as larval development in septic tanks, and to alter diurnal feeding patterns [1, 2]. These characteristics have facilitated the widespread distribution of the vectors and viruses they transmit [3]. Vector control efforts are critical to reduce the health burden of these diseases in endemic areas and to prevent their expansion into new regions. Understanding factors that facilitate the propagation of these important disease vectors is critical to planning surveillance, vector containment, and disease prevention.

The success of *Ae. aegypti* in urban settings has prompted research into the coupled human-natural systems that support their oviposition, development, and survival. *Ae. aegypti* distributions and related disease outbreaks are known to be associated with temperature, humidity, and rainfall/precipitation [4, 5]. *Ae. aegypti* abundance is associated with human-linked factors such as socioeconomic status, water storage, and other aspects of the built environment [6]; however, most research has occurred in humid, tropical climates [7–10]. Investigations in desert contexts are limited. We hypothesize that relationships between human and environmental factors with mosquito abundance and disease transmission risk may differ in magnitude and direction in regions where water resources are scarce and weather conditions are inhospitable.

Climate suitability has constrained the distribution of *Ae. aegypti*, but vulnerable regions are expected to shift in response to climatic change, and possibly urbanization, over the coming decades [11]. Global predictions of *Ae. aegypti* distributions are primarily driven by projected average changes in temperature and rainfall/humidity at coarse spatial scales. Laboratory experiments indicate upper temperature thresholds of 38–42 °C for the development and survival of *Ae. aegypti* which are attenuated with higher humidity [12–14]. Vector population models are sensitive to that upper threshold limit, leading to predictions of population crashes in areas in which temperatures exceed that threshold. Such models prompt hypotheses that *Aedes*-borne disease risk may decrease in areas where climate change will lead to aridification and higher temperatures [15, 16]. However; while intense heat, low rainfall, and low humidity theoretically make desert areas inhospitable to mosquitoes, populations of important vector species, including *Ae. aegypti*, are well established, and transmission of *Aedes*-borne diseases has been reported in desert cities [17]. Although temperatures in the Arizona-Sonora desert region regularly exceed laboratory-derived survival thresholds during summer months, *Ae. aegypti* populations have been detected consistently in Sonoran desert cities like Tucson and Phoenix, Arizona, since their reintroduction to the area more than 25 years ago [18–20]. In 2022, the first locally confirmed case of dengue virus was detected [21]. Not only was there a confirmed human case, but virus-positive mosquites were also discovered. Anthropogenic water sources in arid environments provide aquatic habitat for the immature life stages and are associated with dengue transmission in dry months [22, 23]. The extent to which other built environment factors contribute to *Ae. aegypti* abundance in desert cities remains unclear.

Maricopa County, Arizona, is home to the Phoenix metropolitan area, the sixth largest US city. West Nile virus has been transmitted annually since its introduction in 2003 with sporadic outbreaks [24]. In response, the Maricopa County Vector Control Division (MCVCD) performs weekly surveillance of adult mosquito abundance with a network that now includes over 800 vector traps distributed across the county. This surveillance was heavily utilized during an unprecedented outbreak of West Nile virus in 2021, with over 1400 confirmed cases in Maricopa County alone [25]. The initial focus on *Culex* species was expanded to include adult *Ae. aegypti* mosquitoes. These robust data offered the unique opportunity to investigate predictors of localized *Ae. aegypti* abundance in a large desert city through a secondary data analysis.

Understanding the relative distribution of the mosquitoes across the arid urban environment can identify heterogeneity in risks for epidemics of *Aedes*-borne diseases and guide future vector control recommendations in desert settings. As climate change is predicted to cause widespread aridification, these results may apply more broadly to urban arid environments globally. Our analysis complements a recent study that spatially examined presence/absence of *Ae. aegypti* in Maricopa County [26] to further investigate the coupled human-natural factors that explain heterogeneity in adult *Ae. aegypti* abundance. Given the heat and aridity of the setting, our primary hypothesis was that built water features and non-native landscaping like grass and dense trees support higher adult *Ae. aegypti* abundance by creating microclimate oases in this desert city.

## Methods

### Study site

The study was based in Maricopa County, home to metropolitan Phoenix, with a population of ~ 4.6 million people. Maricopa County falls within the Arizona-Sonora Desert; it is classified as a subtropical desert with seasonal monsoon rainstorms between June and September, with lighter rainfall in December and January. The summer months coincide with extremely high temperatures, with the daily high temperatures commonly exceeding 40 °C and sometimes nearing 48 °C June through September. Native vegetation includes cacti and desert shrubs, but much of the region is bare earth. Maricopa County has one of the fastest growing populations in the US [27], with widespread commercial and residential structures and alterations to the native landscape for both residential and agricultural purposes. Maricopa County has had consistently high incidence of West Nile virus (WNV) since its introduction in 2003. The first confirmed case of locally acquired dengue was identified in November 2022 [21].

### *Aedes aegypti* count data

In response to the endemic WNV threat, the MCVCD undertakes extensive surveillance and control efforts, including weekly mosquito collections from geolocated traps distributed throughout the county (from 765 to 932 unique locations between 2014 and 2017). Although the MCVCD has undertaken surveillance since 2006, the number of traps has increased over the years. This analysis concentrates on the counts of adult female *Ae. aegypti* mosquitoes captured during weekly trapping events between 2014 and 2017, when the number of trap locations had become highly distributed. In densely populated areas, mosquito traps are allocated to blocks of approximately 1 square mile. Most mosquito data from 2014 to 2017 (> 99% of trap collections) were collected using CO_2_-baited Encephalitis Vector Survey (EVS) traps, with BG-Sentinel traps, baited with both BG lures and dry ice, incorporated at a limited number of locations starting in the year 2016 (278 of 38,177 trap-nights [0.73%] in 2016 and 997 of 38,972 trap-nights [2.56%] in 2017). Unpublished experiments run by Maricopa County Vector Control Division and published data by Williams et al. demonstrate that EVS trap counts and relative BG Sentinel trap counts correlate well, allowing relative abundance patterns to be examined using EVS trap data[28]. Traps are set weekly for 24 h. Adult mosquitoes are counted, identified, and pooled for virus testing. Counts exceeding species-specific thresholds–50 or more per trap-night for *Aedes* and 30 or more per trap-night for *Culex*–or any trap pool testing positive for WNV or St. Louise Encephalitis virus trigger targeted insecticide treatments.

### Potential predictor variables

Given the known association between climate and mosquito abundances, all models were adjusted for temperature and rainfall. Estimates of the total precipitation (in mm) and average temperature (in degrees Celsius) at each trap location by month during the study period were obtained from freely accessible estimates of the PRISM Group at Oregon State University at a 4-km grid resolution (https://prism.oregonstate.edu/). Though we had mosquito captures from one night weekly for each trap, these climate data were only available as monthly averages. To account for anticipated delayed impacts on mosquito development, our analysis tested the values lagged from one calendar month prior to the calendar month of the date of collection rather than the month corresponding to the night of collection.

Based on the relatively short expected flight distances of *Ae. aegypti* mosquitoes [29], we analyzed the values of demographic and land cover predictors of interest using a 50-m buffer around each trap. Demographic characteristics of the human population surrounding each trap were estimated from US Census Bureau American Community Survey 5-Year Estimate for the years 2011–2015, based on the 2010 census [30]. Sociodemographic variables including population density, average household size, age, sex, race, income/poverty, and education were calculated as the weighted average value for all census blocks falling within each trap’s 50-m buffer weighted by the percent area covered by each census block. Land cover/land use was estimated based on one-meter resolution aerial images of Maricopa County taken by the National Agriculture Imagery Program (NAIP) during May and June of 2015. The NAIP image included four bands (red, blue, green, and infrared) and enabled calculation of the normalized differential vegetation index (NDVI) [31]. We performed iterative self-organizing (Iso) cluster unsupervised classification in ArcGIS v.9.4 on the four-band plus NDVI composite allowing for 100 potential categories. Two of the authors collaboratively reviewed the output categories against a visual overlay of the original NAIP image to manually reclassify the 100 categories into nine groups: structures, roads/pavement, bare earth, cacti/shrubs, crops/grass, trees, lake/water, swimming pool, and shadow. Grass and crops could not be consistently distinguished from the classification and had to be analyzed collectively. Finally, we used zonal statistics (Tabulate Area 2) to calculate the percentage of each land cover type within the 50-m buffer zone around each trap. The procedure distinguished water and green vegetation from structure, pavement, and bare earth, though visual examination revealed some misclassifications between the latter three categories. Dense tree cover was sometimes misclassified as shadow, though we did not observe instances of shadows being misclassified as tree cover. Models were explored in which shadow and tree cover were included independently or in combination.

### Statistical analyses

Data management and analyses were completed in Statistical Analysis Systems (SAS) version 9.4 (SAS Institute, Cary, NC). The primary outcome of interest was the count of adult female *Ae. aegypti* per night per trap, with most of the traps having repeated measurements on a weekly basis after their introduction. These values were highly skewed—significantly overdispersed and with a highly inflated number of nights where zero *Ae. aegypti* were captured. Simple summary statistics were calculated for all trap-nights occurring within each month of collection to visualize seasonality in figures. We built a multi-level zero-inflated negative binomial (ZINB) regression model to evaluate climate, socioeconomic, demographic, and land cover relationships with *Ae. aegypti* counts using each day of collection as a separate observation, with a random effect to account for repeated observations at the same location. The logistic portion of the ZINB models incorporated the strongest predictive factors: rainfall, temperature, and population density. Crude models for all predictors of counts per trap-night were run in negative binomial and ZINB regression for comparison.

Spline regression was used to fit non-linear associations between the 1-month lagged average temperature and total rainfall and *Ae. aegypti* counts. Rainfall had a generally linear association with count, but temperature had distinct non-linearity, with a positive association increasing to a monthly average temperature of 29 °C and a negative association for increasing temperatures > 29 °C (Additional file 1: Fig. S1). An interaction term was incorporated into all models involving temperature to allow for this threshold effect.

Final model selection considered collinearity between predictors, a priori knowledge about factors influencing *Ae. aegypti* breeding like population density and climate, and the strength of associations from crude ZINB models to build a parsimonious set of covariates of interest. The strongest predictors from crude regression were added first to the ZINB model using *proc genmod*, with additional variables incorporated one by one and retained if they lead to significant improvement of the model fit based on Akaike’s information criterion (AIC). For highly collinear predictors like the various measures of socioeconomic status, we added each to the same base model and retained the one that led to the best fit based on the AIC. The best fit final set of predictors from the fixed effects model was then run in a multi-level ZINB regression using the *proc nlmixed* command with a random error term to adjust for clustering attributable to repeated sampling of the same trap locations [32, 33]. The goodness of fit was assessed for all unique trap locations by comparing the observed relative frequencies of each count with the maximum likelihood estimates of their probabilities [32].

## Results

### Summary of *Ae. aegypti* data

**Fig. 1:**
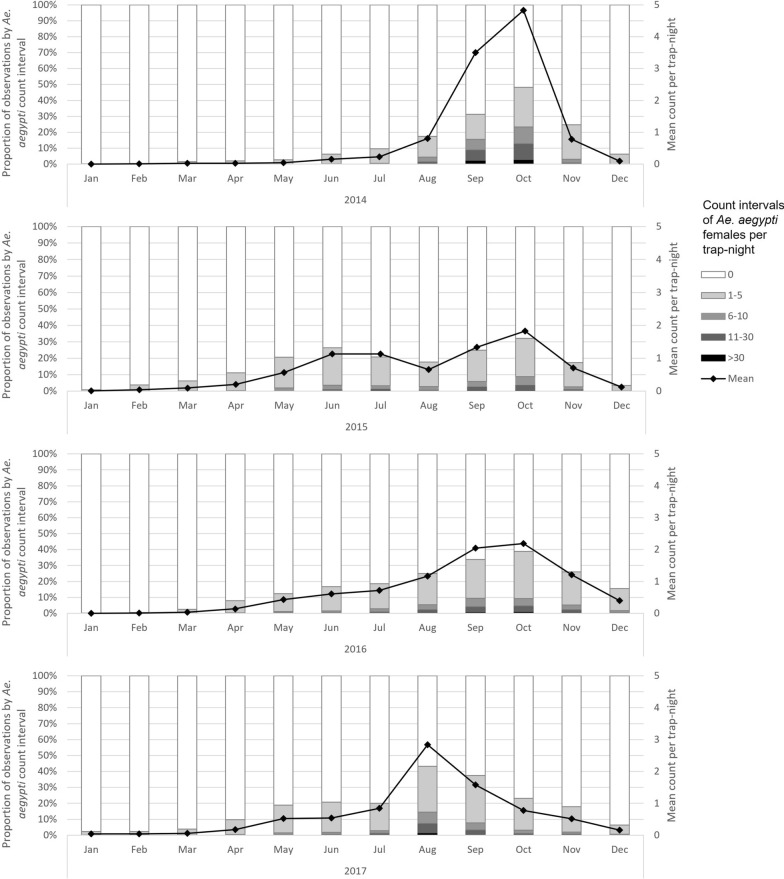
Counts of adult female *Ae. aegypti* mosquitoes captured per trap-night by month, Maricopa County, Arizona, 2014–2017. Intervals for trap counts were manually defined for consistency across months and because of the extremely right skewed nature of the data

**Fig. 2:**
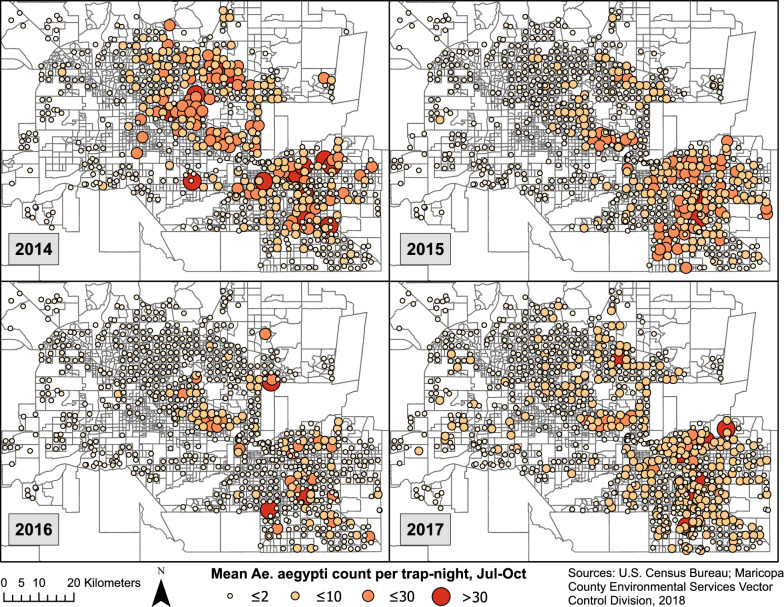
Locations of mosquito traps and average count per trap night during rainy season months (July–October), Maricopa County, 2014–2017

The number of unique trap locations ranged from 765 to 932 between the years 2014 and 2017 (Table 1). Two trap-nights of 139,731 total were extremely high outliers, with counts four and six times the next highest trap-night and approximately 150 and 250 standard deviations above the mean. Because these were thought to be related to unusual hatch events, these two observations were removed from all tables and analyses. More than 109,000 *Ae. aegypti* females were counted from the remaining 139,729 trap-nights. Counts peaked annually between August and October following monsoon rains (Fig. 1). Trap abundance varied spatially during monsoon season (July–October) 2014 to 2017 (Fig. 2). The percent of trap-nights with *Ae. aegypti* female presence increased from 13.6% in 2014 to 18.3% in 2017 (Table 1). Median count per trap-night in positive traps was 2 or 3 each year, but maximums in a trap-night exceeded 200 each year.

**Table 1:** Summary of data on *Aedes aegypti* from Maricopa County surveillance activities, 2014—2017

	Year			
	2014	2015	2016	2017
All traps				
Number of unique trap locations	765	913	932	881
Number of trap-nights	28,131	34,447	38,177	38,972
Total count of adult female *Ae. aegypti*	27,208	24,155	28,986	28,934
Trap-nights with any adult female *Ae. aegypti* present	13.6%	16.3%	16.7%	18.30%
Number of females per trap-night when positive, median (range)	3 (1–215)	2 (1–375)	2 (1–300)	2 (1–325)
EVS traps				
Number of unique trap locations	765	913	914	808
Number of trap-nights	28,131	34,447	37,899	37,975
Total count of adult female *Ae. aegypti*	27,208	24,155	28,732	27,291
Trap-nights with any adult female *Ae. aegypti* present	13.6%	16.3%	16.6%	17.8%
Number of females per trap-night when positive, median (range)	3 (1–215)	2 (1–375)	2 (1–300)	2 (1–325)
BG-Sentinel (Biogents) traps				
Number of unique trap locations	0	0	19	73
Number of trap-nights	0	0	278	997
Total count of adult female *Ae. aegypti*	0	0	254	1,643
Trap-nights with any adult female *Ae. aegypti* present	N/A	N/A	32.4%	40.3%
Number of females per trap-night when positive, median (range)	N/A	N/A	2 (1–20)	2 (1–158)

*EVS* Encephalitis vector survey

### Characteristics of trap locations

Demographic and land cover values surrounding trap locations are summarized in Table 2. Trap locations were distributed throughout Maricopa County, including dense urban areas (population density maximum 26,133 people per square mile) and sparsely populated areas (0.74 people per square mile). The median percent of the population comprised of non-Hispanic white was 70.0%, but ranged from 1.1 to 100%. The median percent of people > 65 years of age was 9.6%, but ranged from 0 to 95.2%. Census blocks were divided into quartiles based on the percent of the block whose income was < 200% of the federal poverty line (range: 0–100%, median 22.5%).

**Table 2:** Demographic and land cover characteristics in the 50 m radius surrounding each mosquito trap under surveillance by the Maricopa County Vector Control Division, 2014—2017

Variable	Median (range)	IQR
Demographic characteristics in census blocks surrounding mosquito traps*		
Population density (people/mile^2^)	3,451.9 (2.85–28,114.5)	1,652.5–5,268.4
Average household size (no. of residents)	2.9 (0–5.5)	2.5–3.4
Median age (years)	36.0 (17.1–77.9)	30.9–42.9
Percent ≥ 65 years old	9.6% (0%–95.2%)	5.9%–15.4%
Percent female	50.6% (2.7%–94.3%)	47.5%–53.7%
Percent non-Hispanic white	70.0% (1.1%–100%)	51.4%–80.7%
Percent adults > 25 years old with only high school degree or less	19.0% (0%–61.0%)	12.3%–28.2%
Percent with limited English proficiency	4.2% (0%–47.9%)	1.7%–10.2%
Median household income (USD)	$65,961.5 ($0–$205,536.0)	$48,056.0–$90,417.0
Percent with income < 200% of poverty level	22.5% (0%–100%)	13.2%–39.7%
Percent vacant houses	9.6% (0%–50.7%)	4.9%–15.3%
Land cover**		
Percent bare earth	21.6% (0%–89.3%)	14.3%–30.5%
Percent grass/crops	17.4% (0%–93.6%)	11.4%–26.6%
Percent trees	6.4% (0%–37.9%)	4.0%–9.6%
Percent cactus/shrub	4.1% (0%–54.6%)	2.8%–5.9%
Percent pool	0.1% (0%–14.3%)	0%–0.5%
Percent lake	0% (0%–32.2%)	0%–0.05%
Percent road/pavement	24.7% (0.2%–85.8%)	17.2%–32.7%
Percent structure	14.3% (0.6%–84.4%)	10.4%–19.4%
Percent shadow	1.4% (0%–13.2%)	0.7%–2.5%

*IQR* interquartile range, *USD* US dollars

*Estimates for traps whose buffer included more than one census block were calculated as an average of the characteristics of overlapping census blocks weighted by the percent contribution of each block to the total area of the buffer zone

**Classified in ArcGIS from 1 m resolution aerial images taken May/June 2015

Traps were often placed in open common areas of neighborhoods, such as parks. The median of grass/crop cover was 17.4% (interquartile range [IQR]: 11.4–26.6%) and that of tree cover was 6.4% (IQR: 4.0–9.6%) in the area within 50 m surrounding traps. Shadow was strongly correlated with tree cover (*r* = 0.75, *P* < 0.0001), which could reflect both creation of shadows by trees and occasional misclassification of dense tree cover as shadow, but it comprised a small amount of ‘land’ cover around the traps, with a median of 1.4% (IQR 0.7–2.5%). Water bodies were also uncommon near traps, with medians of 0.1% for pool cover (IQR: 0–0.5%) and 0% for lake cover (IQR: 0–0.1%). Road, structures, and bare earth covered relatively high proportions of land around the traps compared to the water features and vegetation (Table 2).

### Predictors of *Ae. aegypti* mosquito counts

All exploratory analyses indicated significant overdispersion, so only negative binomial distributions were considered to model the count of *Ae. aegypti* females per trap-night. We incorporated zero-inflation given that 83.6% of all trap-night observations found no *Ae. aegypti* mosquitoes. The logistic portion of the model was built based on a priori knowledge and included the total rainfall in the previous month, the average temperature in the previous month, and the population-density at the trap location [26]. Temperature effects were modeled separately above and below the threshold of 29 °C based on data exploration with spline regression. The zero-inflated negative binomial (ZINB) model was preferred given the notable zero-inflated distribution of the data, though the direction and magnitude of the associations with predictors were largely consistent with those produced by the crude negative binomial models. Adjusted models were built using only ZINB distributions.

Table 3 reports all crude associations between predictors and *Ae. aegypti* counts from the negative binomial portions of the ZINB models as well as the results of the final adjusted model, which included rainfall and temperature lagged from the previous month, population density, average household size, percent of census block that was female, poverty quartile of the census block, the percentage of bare earth within 50 m of the trap, and percentage of tree cover within 50 m of the trap. Average household size and percent female were included in the final ZINB model based on significance in the fixed effects model, though they were not statistically significant after incorporating the random error term for repeated measurements at trap locations in the multi-level adjusted ZINB model (Table 3). The trap-level random error term in the final model was significantly different than 0 (1.93, *P* < 0.0001), suggesting that additional trap-level factors contribute to *Ae. aegypti* variability. Model validation plots indicated a good fit for the frequencies of counts at all trap locations.

**Table 3:** Predictors of adult female *Aedes aegypti* counts by multi-level zero-inflated* negative binomial regression, Maricopa County, Arizona, 2014—2017

Variable (units in regression model)	Crude zero-inflated negative binomial		Adjusted** zero-inflated negative binomial with random error term***	
	IRR (95% CI)	*P*-value	IRR (95% CI)	*P*-value
Climate and other logistical characteristics				
Cumulative rainfall, lagged 1 month (5 mm increase)	1.09 (1.08–1.09)	< 0.0001	1.110 (1.106–1.14)	< 0.0001
Average monthly temperature, lagged 1 month (1 °C increase)				
< 29 °C	1.27 (1.26–1.28)	< 0.0001	1.17 (1.15–1.18)	< 0.0001
≥ 29 °C	0.91 (0.90–0.93)	< 0.0001	0.97 (0.96–0.99)	< 0.0001
Trap type				
Encephalitis vector survey (EVS)	1.0 (ref.)			
BG-Sentinel	1.12 (0.96–1.30)	0.16		
Year				
2014	1.0 (ref.)		1.0 (ref.)	< 0.0001
2015	0.67 (0.63–0.72)	< 0.0001	1.23 (1.16–1.30)	< 0.0001
2016	0.78 (0.73–0.82)	< 0.0001	1.42 (1.32–1.51)	< 0.0001
2017	0.70 (0.66–0.74)	< 0.0001	1.23 (1.15–1.31)	< 0.0001
Land cover				
Percent pool (1% increase)	1.18 (1.13–1.23)	< 0.0001		
Percent lake (1% increase)	0.96 (0.95–0.97)	< 0.0001		
Percent road/pavement (5% increase)	0.997 (0.99–1.01)	0.55		
Percent bare earth (5% increase)	0.84 (0.84–0.85)	< 0.0001	0.94 (0.90–0.96)	< 0.0001
Percent structure (5% increase)	0.88 (0.87–0.90)	< 0.0001		
Percent grass/crops (5% increase)	1.09 (1.08–1.10)	< 0.0001		
Percent trees (1% increase)	1.10 (1.10–1.11)	< 0.0001	1.07 (1.06–1.09)	< 0.0001
Percent cactus/shrubs (1% increase)	0.99 (0.98–0.995)	0.0008		
Percent shadow (1% increase)	1.31 (1.29–1.33)	< 0.0001		
Composite percent grass/crops and trees (5% increase)	1.13 (1.12–1.14)	< 0.0001		
Composite percent trees and shadow (1% increase)	1.08 (1.08—1.09)	< 0.0001		
Demographic characteristics				
Population density (1000 people mile² increase)	1.08 (1.07–1.10)	< 0.0001	1.21 (1.18–1.25)	< 0.0001
Average household size (increase of 1 person)	0.96 (0.93–0.994)	0.019	0.84 (0.73–1.04)	0.012
Median age (5 year increase)	1.03 (1.01–1.04)	< 0.0001		
Percent 65 years or older (10% increase)	0.95 (0.94–0.97)	< 0.0001	0.98 (0.92–1.05)	0.63
Percent female (10% increase)	0.86 (0.83–0.90)	< 0.0001	0.88 (0.76–1.02)	0.079
Percent NHW (10% increase)	1.10 (1.09–1.12)	< 0.0001		
Percent adults > 25 years with high school degree or less (10% increase)	0.80 (0.78–0.81)	< 0.0001		
Percent vacant houses (5% increase)	0.95 (0.93–0.97)	< 0.0001	0.88 (0.80–0.98)	0.015
Percent with limited English proficiency (5% increase)	0.91 (0.89–0.93)	< 0.0001		
Median household income (10,000 USD increase)	1.06 (1.05–1.07)	< 0.0001		
Percent income below 200% of poverty level (10% increase)	0.93 (0.92–0.94)	< 0.0001		
Poverty quartile (based on percent of the census block with income at below 200% of the poverty level)				
Most poverty	1.0 (ref.)		1.0 (ref.)	
Moderate poverty	1.08 (1.02–1.14)	0.005	1.38 (1.10–1.73)	0.0058
Moderately low poverty	1.30 (1.23–1.37)	< 0.0001	1.91 (1.52–2.40)	< 0.0001
Least poverty	1.59 (1.51–1.69)	< 0.0001	2.60 (2.06–3.28)	< 0.0001

*CI * confidence interval, *IRR* incidence rate ratio, *USD* US dollars

*The logistic portion of the zero-inflated models incorporated rainfall lagged 1 month, average temperature lagged 1 month, and population density. Odds ratios for the logistic portion of the final model are included in Additional file 2: Table S1

**Variables included in the final model were those that significantly improved the model fit by Akaike’s information criterion (AIC) after adjustment for other strong predictors. All IRR values are adjusted for the full set of variables included in the column

***A random error term was included in this multi-level model to adjust for repeated measurements at the same trap location

BG-Sentinel traps were positive for at least one *Ae. aegypti* mosquito significantly more often than the EVS traps (38.6% vs. 16.2%, Chi-square *P* < 0.0001, Table 1) but constituted < 1% of trap-night observations. EVS trapping was evenly distributed through the year, but the BG-Sentinel traps were deployed much more often during the rainy season months of July to October (66.8% of BG-Sentinel traps vs. 35.5% of EVS traps, Chi-square *P* < 0.0001). BG-Sentinel traps were associated with higher counts of *Ae. aegypti* than EVS traps, but the difference was not statistically significant in the crude negative binomial portion of the ZINB model after adjusting for temperature, rainfall, and population density in the logistic component (incidence rate ratio [IRR] = 1.12, 95% confidence interval [CI]: 0.96–1.30).

**Fig. 3:**
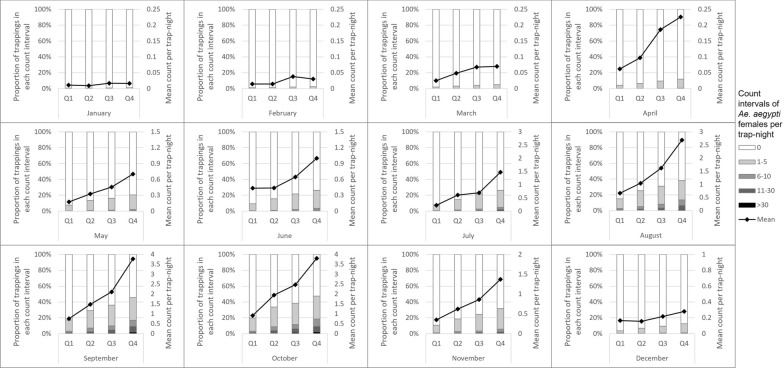
Counts of *Aedes aegypti* females per trap-night by month and quartile of tree cover within 50 m of the trap (Q4 = highest), Maricopa County, 2014–2017. Intervals for trap counts were manually defined for consistency across months and because of the extremely right skewed nature of the data

**Fig. 4:**
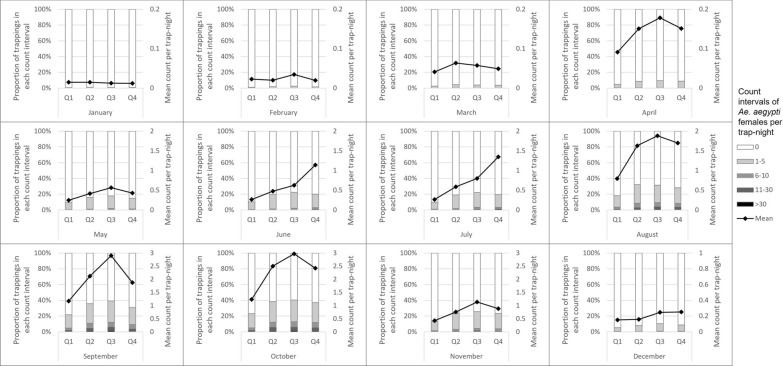
Counts of *Aedes aegypti* females per trap-night by month and quartile of grass cover (Q4 = highest), Maricopa County, 2014–2017. Intervals for trap counts were manually defined for consistency across months and because of the extremely right skewed nature of the data

Counts of *Ae. aegypti* were strongly associated with land cover in the 50-m radius surrounding the traps. The percentage of bare earth around the trap was a strong negative predictor of *Ae. aegypti* counts in the crude ZINB analysis, though the magnitude of the association was weakened in the final adjusted model. An increase of 5% in the amount of bare earth surrounding a trap was associated with a 6% reduction in the counts of *Ae. aegypti* (95% CI: 4–10%). Increasing quartiles of tree cover displayed a clear positive trend with *Ae. aegypti* counts. Differences were greatest during summer monsoon months but extended into November and even weakly into December (Fig. 3). The relationship between grass cover quartile and *Ae. aegypti* counts did not display such a clear trend (Fig. 4). Both were associated with *Ae. aegypti* counts in crude ZINB regression (Table 3), but the association with tree cover was larger and remained significant in the final adjusted model (incidence rate ratio [IRR] = 1.07 for a 1% increase in tree cover, 95% CI: 1.06–1.09). Percent of land cover classified as shadow was also strongly associated with the count of *Ae. aegypti* in the crude ZINB models. Areas classified as shadow on the satellite images were strongly correlated with dense tree cover (*r* = 0.75, *P* < 0.0001). We investigated composite variables that combined tree and shadow percentages as shade cover and tree and grass coverage percentages as vegetation cover, but neither composite variable fit the data better than tree cover alone, and thus these composites were excluded from the final model.

**Fig. 5:**
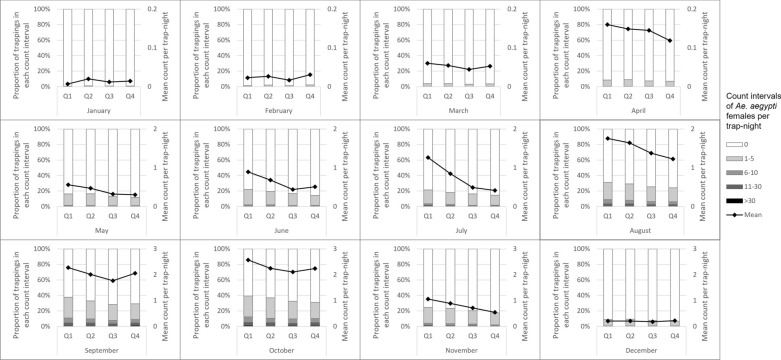
Counts of *Aedes aegypti* females per trap-night by month and quartile of poverty (Q4 = most poverty), Maricopa County, 2014–2017. Intervals for trap counts were manually defined for consistency across months and because of the extremely right skewed nature of the data

Population density was one of the strongest predictors of *Ae. aegypti* counts. In the final adjusted model, *Ae. aegypti* counts were 21% higher with every increase of 1000 people per square mile (95% CI: 18–25%). Census block wealth was positively associated with *Ae. aegypti* counts. Wealthier areas had higher counts than census blocks with larger proportions of residents living in households below 200% of the federal poverty line. Population density confounded the crude relationship between wealth and *Ae. aegypti* counts toward null, i.e. reducing the magnitude of the crude association compared to the association after adjusting for population density, as less dense suburban areas tended to be wealthier (correlation between population density and percent living below 200% of the federal poverty line, *r* = 0.364, *P* < 0.0001). The relationship was reflected most clearly in an earlier and steeper seasonal increase in *Ae. aegypti* counts for traps in the wealthiest quartile of census blocks (Fig. 5). Tree cover and grass cover tended to be significantly higher in wealthier census blocks (p for both correlations < 0.0001) and explained part of the association between wealth and *Ae. aegypti* counts; however, the association with wealth was highly significant even in the final model that adjusted for tree cover, with the wealthiest quartile of census blocks having 2.60 times the counts of *Ae. aegypti* as blocks in the quartile with highest poverty levels (95% CI: 2.06–3.28).

## Discussion

Despite exceedingly high temperatures and aridity, *Ae. aegypti* populations are abundant in developed urban areas of Maricopa County (Phoenix), Arizona. Coupled human-natural factors explained spatial heterogeneity in counts of female *Ae. aegypti* mosquitoes even after accounting for variability in rainfall and temperature. Counts of female *Ae. aegypti* mosquitoes in this desert city most notably increased with human population density, census block wealth, and nearby tree cover. These factors contributed to seasonal increases in *Ae. aegypti* counts that started earlier in the year, lasted later, and peaked with greater abundance. Our findings differ from relationships reported in other climates, as described below, and have important implications for *Ae. aegypti* control strategies in desert cities.

These results suggest that *Ae. aegypti* abundance in Maricopa County was not absent at the temperature thresholds previously identified in laboratory experiments [12, 13]. Depending on the life stage of the mosquito, such experiments have found *Ae. aegypti* mortality to increase above 38–42 °C; even brief laboratory exposure (15–30 min) to temperatures > 42 °C is typically highly fatal to adults and to > 43.3 °C (110°F) is universally fatal [12, 13]. Weather stations in urban central locations of Maricopa County regularly measured daytime temperatures that exceeded 43.3 °C in the summer months of 2014 to 2017, with temperatures up to 49 °C being detected [34]. The strikingly high counts of *Ae. aegypti* mosquitoes during and immediately after these hot summer months defy anticipated heat impacts. Recent modeling efforts using the Dynamic Mosquito Simulation Model (DyMSiM) found that model predictions are sensitive to the temperatures chosen as the upper threshold for survival, particularly in areas at the margins of these thresholds like the Arizona-Sonora Desert [16]. Climate change-driven increases in temperature and aridity may only reduce *Aedes*-borne virus transmission potential after surpassing higher thresholds than those derived from laboratory experiments. These complex associations between human modification of the environment to maintain thermal comfort and the indirect impact on suitability for *Ae. aegypti* survival need more exploration to better parameterize climate change projection models.

The persistent abundance of *Ae. aegypti* in Maricopa County may be explained by biological adaptations promoting heat tolerance, behavioral tendencies to exploit microclimate oases with more favorable conditions, or a combination of the two [35]. The exact mechanisms driving the relationships between land use/landcover and *Ae. aegypti* abundance are likely moderated by temperature, humidity, water sources, and food sources. As noted in previous work, higher humidity associated with monsoons could blunt the impact of these high temperatures, but our model adjusted for rainfall and that explanation does not fully account for rising abundance as early as June and July, prior to monsoon rainfall [14]. While larger scale climate metrics predict mosquito population dynamics, several studies have noted that microclimatic variations may be most relevant to mosquito survival [4, 7, 8]. In Phoenix, and other locations, vegetation significantly reduces day and nighttime ambient temperature [36]. Our results demonstrate a clear positive trend between tree cover and mosquito abundance. The strong association between increasing land cover by dense trees and trap counts of *Ae. aegypti* and the weaker association with grass/crops suggest that transformation of native desert land through increased vegetation and landscaping may create favorable microclimates. Such landscaping requires added water, potentially increasing oviposition sites and increasing local humidity. Dense tree cover also creates shade, which modulates temperature significantly [36]. In a few preliminary observations with thermal imaging cameras, we observed daytime temperature gradients of > 10 °C between bare earth/pavement and shaded areas. The strong crude association between land cover classified as shadow and *Ae. aegypti* counts provides further support for this mechanism, as does a recent study from tropical Machala, Ecuador, which found patios with shade to be associated with dengue incidence [37]. Further research into the specific mechanisms by which vegetation promotes *Ae. aegypti* abundance in the desert would be useful to inform control efforts, which might include replacement or selection of specific tree species, improved drainage for oviposition site reduction, pesticide spraying in targeted landscaping, or an overall shift to xeriscaping.

Maricopa County identified its first case of locally acquired dengue in November 2022 illustrating the potential for *Aedes*-borne disease outbreaks in the county [21]. Notable dengue and chikungunya outbreaks were reported within the Arizona-Sonora desert region, including a binational outbreak in the US-Mexico border town of San Luis Rio Colorado, Sonora [38]. Previous binational studies indicate socioeconomic and cultural differences that impact vector-human contact may partially explain this discrepancy [39, 40]. Our study identified higher abundance in higher income neighborhoods which may also translate into lower transmission risk because of reduced vector-human contact. We are limited by outdoor trap collections. A recent study in LA County indicated higher indoor abundance was associated with lack of AC and screens on doors and windows [6].

Several of our findings about predictors of *Ae. aegypti* abundance mirror those of previously published literature. Given their importance to biological development, breeding, and survival, temperature and precipitation are widely linked to the abundance of *Ae. aegypti*, whether larvae or adults [7, 41–45]. We found human population density to be another strong predictor of *Ae. aegypti* presence and count in Maricopa County, similar to earlier studies that have linked *Ae. aegypti* with metrics of urbanicity/human residence [10, 26, 41, 42, 46, 47]. This corresponds with the highly anthropophilic nature of the species, particularly in this setting where the native desert ecosystem is inhospitable.

Alternatively, the associations with vegetation are context-specific. Vegetation and tree cover have also been found to be positively associated with *Ae. aegypti* presence or abundance in a few other desert cities [26, 45, 48], whereas the opposite has been reported from cities in more tropical or temperate zones [7, 8, 46]. These differences are reasonable if tree cover does not play a major role in creating habitable microclimates in those contexts, and instead the greater density of natural vegetation simply reflects lower degrees of urban development/human residence, as suggested by Lorenz et al*.* [46]. This distinction emphasized the limited generalizability of such findings and the need for research across a variety of climatic and cultural settings to inform best practices for local vector management.

The finding that *Ae. aegypti* counts were higher in wealthier census blocks in Maricopa County, Arizona, also differed from other settings where *Ae. aegypti* counts and dengue risks have been higher in poorer areas [6, 10, 38, 46]. This association was partially explained by the higher density of non-native vegetation (grass and trees) we observed in wealthier blocks in Phoenix, but the association with socioeconomic status persisted even after adjustment for land cover in the final model. Additional research should evaluate whether the positive association between wealth and *Ae. aegypti* counts in Maricopa reflect residual confounding from land cover microclimate effects, possibly through increased water use and micro-variability in humidity or whether other mechanisms play a role.

This study complements a recently published analysis of MCVCD *Ae. aegypti* data. Holeva-Eklund et al. utilized a Maxent species distribution model to identify locations in Maricopa County that are consistently suitable for *Ae. aegypti* presence, examining socioeconomic predictors, temperature, precipitation, and elevation [26]. Our model adds to theirs by evaluating the predictors of abundance using detailed high resolution satellite imagery to characterize specific habitat characteristics proximal to trapping locations after accounting for key presence-absence predictors (temperature, rainfall, and population density) in a ZINB model. They similarly found population density to be an important predictor for habitat suitability but found median income to be a weak predictor. In our analysis, we observed that socioeconomic status indicators, including the proportion of people living below 200% of the poverty line from the final model, were significantly associated with *Ae. aegypti* counts. The relationship was confounded toward the null by population density, as wealthier neighborhoods in the metropolitan area tended to be less dense, and was stronger in the adjusted final model than the crude ZINB model for that reason.

These analyses utilized an extremely large, systematic mosquito surveillance dataset, linked to high-resolution land cover and sociodemographic data, and give insight into abundance of this critical vector species in an understudied desert region. However, as a secondary analysis of data collected for other purposes, our study had several limitations. First, the vast majority of the *Ae. aegypti* count data came from EVS traps, which are not as effective as BG traps for capturing *Ae. aegypti* mosquitoes[28]. Trap type was not a significant predictor of *Ae. aegypti* counts in the final adjusted model, although this was likely due to the relatively small number of data points from BG-Sentinel traps (< 1%). Validity of trap type comparisons was further limited by the exploratory rather than systematic nature of BG-Sentinel trap deployment, with BG traps being introduced only in 2016, having approximately twice as many observations during rainy season months as dry season months, and being placed responsively after exceedances of *Ae. aegypti* in EVS traps, all likely factors contributing to relatively high counts. Second, temperature and rainfall were the only time-varying predictors in our analysis, with census estimates derived from a 2015 projection and land cover classified based on an image from early summer 2015. This could lead to some misclassification, particularly in areas of the city that were undergoing urbanization. Temperature and rainfall data were also only available on a monthly scale and could not be lagged 30 days from each individual trap-night observation, so they represented coarse adjustments in the modeling. Third, the location of traps within blocks was not random, tended to be near open public areas like parks, and may have been associated with wealth. By relying on socioeconomic variables at the census block level, our results may be affected by arbitrary census block lines (Modifiable Areal Unit Problem). Fourth, though it enabled the county-wide scale of the analysis, the unsupervised classification method was imperfect for delineating land cover. The program could not reliably distinguish grass from agricultural crops, so the two had to be assessed jointly. Very dense tree cover was also noted to be misclassified as shadow on visual examination. Though we did not quantify these misclassifications, shadow, trees, and grass/crops appeared to be reliably distinguished from other surface types–water sources, structures, roads, shrub/cacti, and bare earth–by visual examination. Since the classification was automated for the entire study area, errors are unlikely to be biased by the *Ae. aegypti* abundance within the buffer zones and therefore should limit the precision of the estimated associations for each of the three categories rather than creating the appearance of associations that do not truly exist. Finally, this analysis concentrated on specific hypotheses with a fairly limited set of predictors, some quite coarse in their temporal and spatial scales, and there was considerable heterogeneity in the data unaccounted for in the final model, leaving fertile ground for future research. While this analysis of secondary data carries limitations for detailed exploration of survival mechanisms, the large geographic area covered and the high frequency of trap-nights provided a rich dataset in an understudied environment that demonstrates that *Ae. aegypti* mosquitoes can be abundant in urbanized desert settings.

In an effort to mitigate the impact of urban heat islands, Maricopa County set a goal of increasing tree cover from 10% in 2010 to 25% by 2030 [49]. While increasing vegetation demonstrably reduces ambient temperature, a critical need in the face of increasing extreme heat [36], it may have unintended consequences by increasing vector densities. Additional research is being undertaken to understand the relationships between vegetation cover and the most immediate mosquito-borne disease threat to Maricopa County, West Nile virus. Despite mosquito-borne disease risk, heat currently is a larger public health burden, with the deaths of 172 people in Phoenix in 2017 attributed to extreme heat [50], and with the burden expected to increase with climate change [51]. Additional analyses to determine the specific causal link between tree cover and *Ae. aegypti* abundance (tree height, density of shade cover, choice of tree species) could provide options to adapt to increasing heat while minimizing the unintended consequences of increased vector density. However, our results suggest that surveillance and vector-control measures should be supplemented in areas with tree cover to minimize the risk for local *Aedes*-borne virus transmission.

## Conclusions

Despite the exceedingly high temperatures and aridity of the desert climate, *Ae. aegypti* populations are abundant in urban areas of Maricopa County (Phoenix), Arizona, particularly during the summer monsoon season when rainfall is markedly increased, and temperatures are often > 38 °C. Coupled human-natural factors explained spatial heterogeneity in counts of female *Ae. aegypti* mosquitoes even after accounting for variability in rainfall and temperature. Counts of female *Ae. aegypti* mosquitoes in this desert city most notably increased with human population density, census block wealth, and nearby tree cover. These factors contributed to seasonal increases in *Ae. aegypti* counts that started earlier in the year, lasted later, and peaked with higher abundance. These findings differ from relationships reported in other climates and have important implications for projections of arboviral disease risk and *Ae. aegypti* control strategies in desert cities.

### Supplementary Information

**Additional file 1: Figure S1.** Spline regression of predicted probability of *Aedes aegypti* presence by climate variables, **A** rainfall, and **B** temperature.

**Additional file 2: Table S1.** Odds ratios of the logistic component of the final adjusted zero-inflated negative binomial model.

## Data Availability

The datasets used and/or analyzed during the current study are available from the MCVCD and corresponding author on reasonable request.
