# Supplementary material for: Aedes aegypti abundance in urban neighborhoods of Maricopa County, Arizona, is linked to increasing socioeconomic status and tree cover
Source: Parasit Vectors. 2023 Oct 8;16:351. doi: 10.1186/s13071-023-05966-z (PMC10560435; doi:10.1186/s13071-023-05966-z)
Supplement: Supplementary file 1 — Additional file 1: Figure S1. Spline regression of predicted probability of Aedes aegypti presence by climate variables, A rainfall, and B temperature. [file 13071_2023_5966_MOESM1_ESM.pptx]

## Slide 1
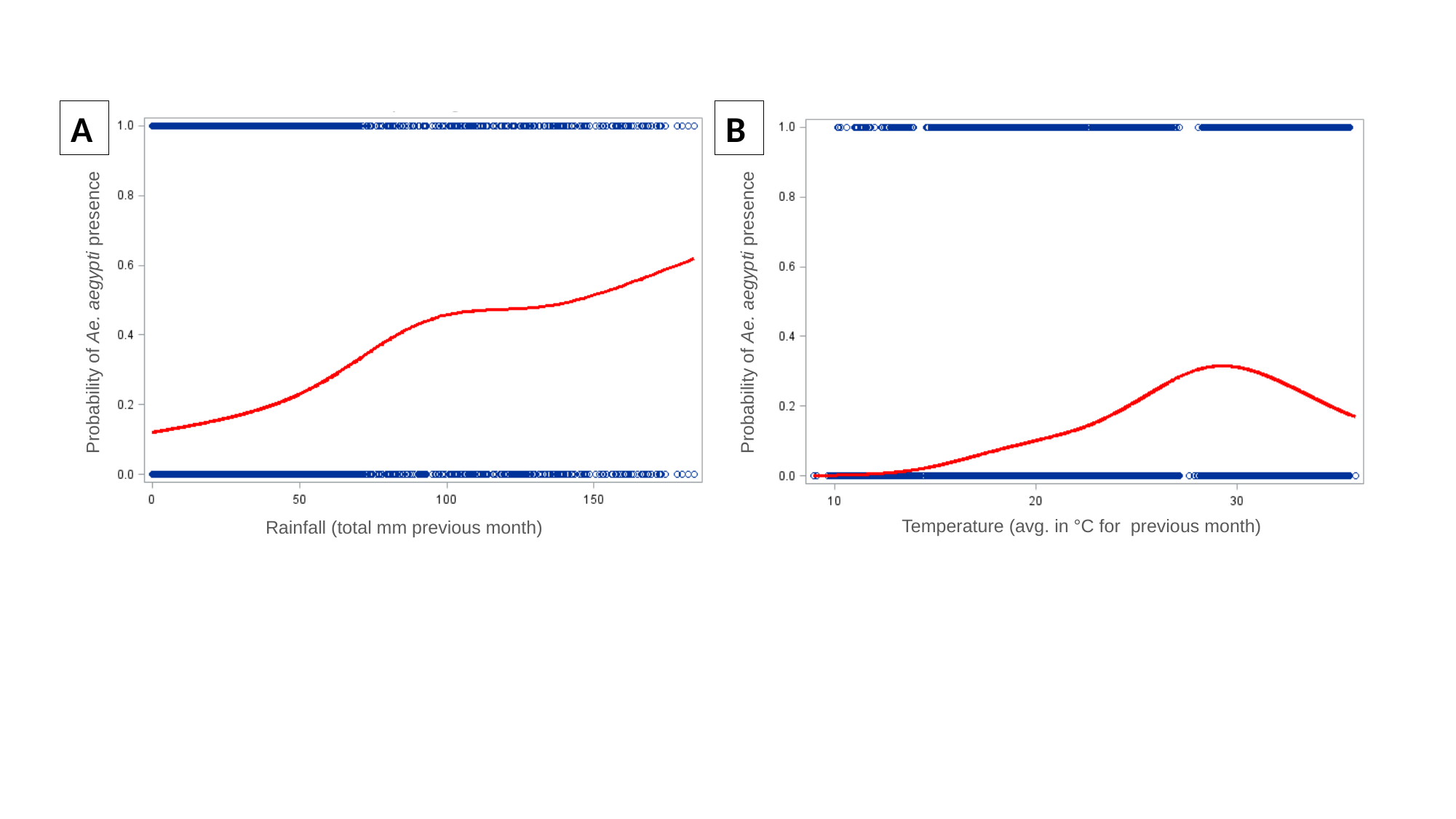

A
Rainfall (total mm previous month)
Probability of Ae. aegypti presence
B
Probability of Ae. aegypti presence
Temperature (avg. in °C for previous month)
