# Supplementary material for: Aedes aegypti abundance in urban neighborhoods of Maricopa County, Arizona, is linked to increasing socioeconomic status and tree cover
Source: Parasit Vectors. 2023 Oct 8;16:351. doi: 10.1186/s13071-023-05966-z (PMC10560435; doi:10.1186/s13071-023-05966-z)
Supplement: Supplementary file 2 — Additional file 2: Table S1. Odds ratios of the logistic component of the final adjusted zero-inflated negative binomial model. [file 13071_2023_5966_MOESM2_ESM.docx]

**Additional file 2: Table S1. Odds ratios of the logistic component of the final adjusted zero-inflated negative binomial model**

| **Variable (units in regression model)** | **Logistic components of the final adjusted zero-inflated negative binomial model** | |
| --- | --- | --- |
|  | **OR (95% CI)** | **p-value** |
| Cumulative Rainfall, lagged 1 month (5 mm increase) | 1.061 (1.058 – 1.064) | <0.0001 |
| Average Monthly Temperature, lagged 1 month (1°C increase)       <29°C       ≥29°C | 1.222 (1.215 – 1.228)  0.960 (0.948 – 0.972) | <0.0001  <0.0001 |
| Population density (1000 people/sq mile increase) | 1.107 (1.101 – 1.112) | <0.0001 |
| CI = Confidence interval; OR = Odds ratio | | |
